# The Big Five, Aesthetic Judgment Styles, and Art Interest

**DOI:** 10.5964/ejop.v14i4.1479

**Published:** 2018-11-30

**Authors:** Reza Afhami, Shahin Mohammadi-Zarghan

**Affiliations:** a Tarbiat Modares University, Tehran, Iran; Department of Psychology, Webster University Geneva, Geneva, Switzerland; The Maria Grzegorzewska University, Warsaw, Poland

**Keywords:** personality, Big Five, aesthetic judgment style, openness to experience, art interest

## Abstract

The current study aimed to examine the associations between the Big Five personality dimensions, aesthetic judgment styles, and art interest. Participants (N = 253) were university students in Tehran, Iran. All participants completed measures of personality, aesthetic Judgment styles, and general interest in art. Results suggested that Openness to Experience was related to advanced styles of art judgment and interest in art-related activities. Regression analyses showed that sex (β = .18, t = 3.18, p = .002), Emotional Stability (β = .14, t = 2.47, p = .01), Openness to Experience (β = .18, t = 3.14, p = .002), symbolic aesthetic judgment style (β = .31, t = 4.63, p < .001), and concrete aesthetic judgment style (β = -.19, t = -3.25, p = .001) significantly predicted art interest. The role of personality and individual difference constructs in aesthetic judgment and art interest is discussed and future directions are outlined.

I acquired a strong taste for music, and used very often to time my walks so as to hear on weekdays the anthem in King’s College Chapel. This gave me intense pleasure, so that my backbone would sometimes shiver. (Charles Darwin)

Social scientists have shown interest in scientific examination of art and aesthetic experiences. Personality psychologists, in particular, have been increasingly paying attention to personality traits and emotional factors that can predict art-related behavior (see Habibi & Damasio, 2014). The universal aspects of art across cultures make aesthetic experiences an important aspect of human life that is both reflected in and predicted by personality traits across cultures (De Smedt & De Cruz, 2010). The present work examines the role of aesthetic judgment styles and normative personality traits in predicting general interest in art in a non-Western culture. Such investigation can potentially uncover the incremental role of aesthetic judgment styles in predicting scores on art interest scales.

The Five-Factor Model (FFM) or Big Five may be considered the most widely used taxonomy of personality traits. This comprehensive model was developed empirically by examining patterns of associations among trait descriptors (John, Naumann, & Soto, 2008). Statistical analyses suggest that *five* factors underlie the structure of human personality trait descriptors. Some debate still exists on construing and labeling the emerged factors; however, the strongest debate has surrounded the interpretation of the fifth factor, which has been explained in terms of Culture, Intellect, Openness to Experience, and Imagination. The most widely used label for the fifth factor is Openness to Experience, or sometimes abbreviated as Openness. Yet, some scholars prefer to use Openness/Intellect, contending that Openness to Experience and Intellect are equally important aspects of the broader factor (Saucier, 1992). In lexical studies, Intellect is reflected by adjectives such as *intellectual*, *intelligent*, *clever*, and *philosophical*. Openness, on the other hand, is reflected by adjectives such as *artistic*, *perceptive*, *poetic*, and *fantasy-prone*. Saucier (1994) proposed “Imagination” as an alternative label for the Openness/Intellect factor, as imagination can be manifest both intellectually and aesthetically (see Nusbaum & Silvia, 2011)

Since Openness to Experience consists of creativity- and art-related facets, art researchers have shown increasing interest in this basic factor of personality (Feist, 1998; McCrae, 2007; Silvia & Nusbaum, 2011). Open individuals are not the passive recipients of experiences. Openness to Experience involves motivation, needs for variety (Maddi & Berne, 1964), cognition (Osberg, 1987), and understanding. This active pursuit of experience can be fully understood in open individuals’ active curiosity, especially in aesthetic contexts. The clearest evidence of open individuals’ need for experience is evident in their appreciation of the arts. Thus, psychology of creativity, aesthetics, and art is more related to this personality factor (DeYoung, Quilty, Peterson, & Gray, 2014; Furnham & Chamorro-Premuzic, 2004) rather than other Big Five dimensions (i.e., Extraversion, Agreeableness, Conscientiousness, and Neuroticism). Obviously, some studies have reported significant associations between other Big Five dimensions and aesthetic experiences (e.g., Swami, Stieger, Pietschnig, & Voracek, 2010). Specifically, Agreeableness and Conscientiousness have regularly shown weak negative correlations with art interests, activities, and knowledge. On the other hand, Extraversion and Neuroticism have usually shown weak positive correlations with these art-related variables (Furnham & Chamorro-Premuzic, 2004). Therefore, it is important to consider all five factors of personality in examination of aesthetic experiences.

An increasing number of studies have begun to investigate the associations between the Big Five, art interest, and aesthetic experiences. Furnham and Chamorro-Premuzic (2004) examined the relationships between personality, intelligence, art interest, and art judgment. These authors found a positive association between Openness to Experience and art experience (i.e., art preferences), but not art judgment (i.e., ability). Interestingly, intelligence was significantly associated with art judgment, but not with art experience. Additionally, art judgment was correlated with Extraversion and lower levels of Conscientiousness. Another study (Chamorro-Premuzic & Furnham, 2004) reported a positive association between Openness to Experience and art activities (e.g., visiting art galleries and purchasing artworks). Higher Openness to Experience and lower Extraversion predicted art knowledge. Finally, low Conscientiousness, low Extraversion, art interest, and intelligence predicted art judgment (ability).

Psychologists have recently shown increasing interest in the utility of intelligence (as mental ability) and personality (as repeated styles of behavior and cognition) in predicting aesthetic experience. For example, visual aesthetic sensitivity has been historically conceived as an intelligence-independent and personality-independent disposition (see Frois & Eysenck, 1995). Yet, recent studies suggest that aesthetic experience can be predicted by personality traits (Furnham & Chamorro-Premuzic, 2004; McCrae, 2007; Rawlings, Barrantes-Vidal, & Furnham, 2000) and is cognitively facilitated (Leder, Belke, Oeberst, & Augustin, 2004; Reber, Schwarz, & Winkielman, 2004; Silvia, 2006). More recently, Myszkowski, Storme, Zenasni, and Lubart (2014) examined the Visual Aesthetic Sensitivity Test in young adults samples and found it to be predicted by intelligence, openness to aesthetics, and divergent thinking.

The above-mentioned studies relied on the conceptualization of artistic judgment as a measure of ability (see Myszkowski, Storme, & Zenasni, 2016). In fact, there have been a number of studies on constructing and validating tests of artistic judgment (e.g., Eysenck & Castle, 1971; Graves, 1948; Stallings & Anderson, 1969). A more recent model of aesthetic judgment has been proposed by Bahrami-Ehsan, Mohammadi-Zarghan, and Atari (2015). According to these authors, individuals have different *styles* of judging art and these aesthetic styles are significantly associated with cognitive styles and thinking styles. In other words, individuals perceive, evaluate, and experience art differently (see Axelsson, 2007). This four-factor model of styles in aesthetic judgment posits that individuals tend to react to artworks differently and their reactions may be categorized into one of the following styles: *concrete, analytical, symbolic*, and *emotional*. Concrete judgment style is typically considered a less developed/professional style of reacting to art. On the contrary, emotional aesthetic judgment is considered the most advanced style of reacting to art. These authors further proposed that aesthetic judgment styles have developmental roots and get more advanced as one masters more advanced cognitive faculties. Pourhosein, Mohammadi-Zarghan, Soufiabadi, and Atari (2017) related ego development (Loevinger, 1976) to aesthetic judgment styles and concluded that concrete style is the least advanced one, with emotional style being the most advanced one.

The present study aimed to examine the associations between the Big Five dimensions of personality, aesthetic judgment styles, and interest in art. We hypothesized that Openness to Experience would be positively associated with emotional aesthetic judgment style (H1) and negatively associated with concrete aesthetic judgment style (H2). Openness to Experience was also hypothesized to be positively correlated with art interest (H3). Further, we hypothesized that emotional aesthetic judgment style would be positively associated with art interest (H4) and that concrete aesthetic judgment style would be negatively associated with art interest (H5). Finally, we conducted a hierarchical regression analysis to examine the unique roles of the Big Five personality dimensions and aesthetic judgment styles in predicting art interest.

## Methods

### Participants and Procedures

We recruited 253 individuals (54.9% women) from university settings in Tehran, Iran. The mean age of the participants was 24.4 years (*SD* = 5.5 years). The majority of the participants (68.8%) were students in art-related fields. All participants were invited to take part in a psychological study regarding psychology and art. Upon agreement, participants completed paper-and-pencil versions of a number of measures. Of note, participants were not compensated.

### Measures

#### The Big Five

The Ten-Item Personality Inventory (TIPI; Gosling, Rentfrow, & Swann, 2003) is a very short measure of the Big Five. Each dimension (i.e., Extraversion, Agreeableness, Conscientiousness, Emotional Stability, and Openness to Experience) is measured with two items, one of which requires reverse scoring. Items are rated along a 7-point scale ranging from 1 (*Strongly disagree*) to 7 (*Strongly agree*). The TIPI has shown adequate psychometric properties across cultures (Oshio, Abe, Cutrone, & Gosling, 2013; Storme, Tavani, & Myszkowski, 2016). The Persian translation of the TIPI has been used in previous studies in Iran (Atari & Yaghoubirad, 2016). The internal consistency coefficients (Cronbach’s alphas) for Extraversion, Agreeableness, Conscientiousness, Emotional Stability, and Openness to Experience were .53, .21, .50, .37, and .34, respectively. As noted by Gosling et al. (2003) internal consistency coefficient is not a good way to assess two-item scales’ reliability. The present alpha coefficients are close to those of the Persian TIPI’s subscales previously reported by Atari, Barbaro, Sela, Shackelford, and Chegeni (2017).

#### Aesthetic Judgment Styles

We used the Aesthetic Judgment Style Scale (AJSS; Bahrami-Ehsan et al., 2015) to measure each participant’s style in response to artworks. The AJSS is a 32-item scale with adequate reliability and validity. The AJSS measures four aesthetic judgment styles: Concrete (sample item: “I prefer to observe artworks, rather than discussing them”), analytical (sample item: “I tend to assess artworks logically”), symbolic (sample item: “I enjoy reasoning about latent symbols in artworks”), and emotional (sample item: “Each time I see my favorite artist’s artworks, I feel closer to them”). Each subscale consists of 8 items. We averaged all 8 items to reach a total score on each subscale. All items are rated along a 4-point scale (1 = *Completely disagree*, 4 = *Completely agree*). In the present sample, internal consistency coefficients were satisfactory. Specifically, Cronbach’s α coefficients were .68, .76, .77, and .77 for concrete, analytical, symbolic, and emotional styles, respectively.

#### Art Interest

We used the following questions to measure participants’ general interest in art (see Chatterjee, Widick, Sternschein, Smith, & Bromberger, 2010; Tschacher, Bergomi, & Tröndle, 2015): (1) How much are you familiar with art schools and movements?, (2) How much information do you have regarding art in general?, (3) How much are you familiar with important artists?, (4) How regularly do you visit art galleries?, and (5) How regularly do you take part in art-related classes and workshops?. All questions were rated on a 4-point scale ranging from 1 (*Never*) to 4 (*Very much*). A principal-axis exploratory factor analysis (EFA) suggested a unidimensional structure (KMO = .83, Bartlett’s χ^2^ [10] = 580.34, *p* < .01, λ_1_ = 3.20, item-factor loadings = .65 - .86), explaining 63.97 percent of the total variance. Face validity of these items were initially confirmed by an independent panel of two art professionals. This scale was internally consistent in the current sample (Cronbach’s α = .85).

### Statistical Analysis

We categorized our analytic strategy into three consecutive steps. In the first step, descriptive statistics and sex differences in all study variables were examined. In the second step, bivariate correlational analyses were conducted. Finally, we conducted a linear hierarchical regression analysis to predict participants’ interest in art according to their personality and aesthetic judgment styles. In the first block, we included personality dimensions. In the second block, we included aesthetic judgment styles. All analyses were conducted using SPSS 22.0.

## Results

### Descriptive Analyses

All descriptive statistics for men and women are presented in Table 1. As shown in Table 1, women were more interested in art compared to men (*t* = 4.14, *p* < .001, *d* = 0.51). Men scored significantly higher on concrete aesthetic judgment style (*t* = -2.02, *p* = .044, *d* = 0.27). Women scored higher on Agreeableness (*t* = 3.34, *p* = .001, *d* = 0.41). All sex differences were small to moderate in magnitude, with art interest showing the largest effect size (*d* = 0.51). Further, we examined all study variables across art (*n* = 174) and non-art (*n* = 78) students. One student had not indicated his/her field of study and was excluded from this analysis. Art students scored significantly higher on art interest (*t* = 9.75, *p* < .001, *d* = 1.13), symbolic aesthetic judgment style (*t* = 3.59, *p* < .001, *d* = 0.49), and Agreeableness (*t* = 2.29, *p* = .02, *d* = 0.31). Non-art students, on the other hand, scored significantly higher on concrete aesthetic judgment style (*t* = 3.87, *p* < .001, *d* = 0.53). Finally, we examined the associations between study variables and age. Age was positively correlated with symbolic (*r* = .18, *p* < .01) and analytical (*r* = .22, *p* < .01) styles of judgment in artistic contexts. Moreover, a significant, yet small-sized, correlation emerged between age and Conscientiousness (*r* = .13, *p* = .04).

**Table 1:** Descriptive Statistics and Sex Differences in All Study Variables

Variable	Men (*n* = 114)		Women (*n* = 139)		Sex effect		
	*M*	*SD*	*M*	*SD*	*t*	*p*	*d*
Art interest	2.27	0.71	2.62	0.63	4.14	< .001	0.51
Emotional AJS	3.04	0.55	3.12	0.58	1.10	.274	0.14
Symbolic AJS	2.70	0.54	2.84	0.57	1.94	.054	0.25
Analytical AJS	2.81	0.53	2.74	0.58	-0.99	.323	0.13
Concrete AJS	2.46	0.58	2.31	0.53	-2.02	.044	0.27
Extraversion	4.18	1.53	4.54	1.58	1.80	.073	0.23
Agreeableness	4.35	0.97	4.81	1.19	3.34	.001	0.41
Conscientiousness	5.33	1.26	5.26	1.46	-0.41	.683	0.05
Emotional Stability	4.11	1.40	3.75	1.61	-1.91	.057	0.24
Openness to Experience	5.36	1.29	5.51	1.17	1.03	.306	0.12

*Note*. AJS = Aesthetic Judgment Style.

### Correlational Analysis

The correlation coefficients between the study variables are presented in Table 2. As can be seen, Openness to Experience was positively associated with emotional aesthetic judgment style (*r* = .19, *p* < .01), while negatively correlated with concrete aesthetic judgment style (*r* = -.26, *p* < .01). Therefore, Hypotheses 1 and 2 are supported. Openness to Experience was also significantly positively associated with interest in art (*r* = .28, *p* < .01), lending support to Hypothesis 3. Emotional aesthetic judgment style was positively associated with art interest (*r* = .18, *p* < .01). Therefore, Hypothesis 4 is fully supported. Finally, a negative correlation emerged between concrete aesthetic judgment style and art interest (*r* = -.27, *p* < .01). Thus, Hypothesis 5 is supported by the correlational analysis.

**Table 2:** Associations Between Personality Dimensions, Aesthetic Judgment Styles, and Art Interest

Variable	1	2	3	4	5	6	7	8	9
1. Art interest	–								
2. Emotional AJS	.18**	–							
3. Symbolic AJS	.40**	.29**	–						
4. Analytical AJS	.19**	.18**	.52**	–					
5. Concrete AJS	-.27**	.04	-.08	.14*	–				
6. Extraversion	.05	.01	.04	-.02	.02	–			
7. Agreeableness	.03	-.01	-.06	-.08	.11	-.07	–		
8. Conscientiousness	-.03	-.01	.07	.07	.01	.06	.02	–	
9. Emotional Stability	.15*	.03	.11	.06	.04	.05	.01	.16*	–
10. Openness to Experience	.28**	.19**	.05	.11	-.26**	.06	-.07	.04	.09

*Note*. AJS = Aesthetic Judgment Style.

*p < .05. **p < .01.

Among other correlations, Emotional Stability was significantly positively correlated with art interest (*r* = .15, *p* < .05). Regarding aesthetic judgment styles, symbolic aesthetic judgment style emerged as a very strong correlate of art interest (*r* = .40, *p* < .01). Analytical aesthetic judgment style was also significantly positively associated with art interest (*r* = .19, *p* < .01).

### Regression Analysis

As mentioned, in this step we conducted a linear, two-block, hierarchical regression analysis onto art interest. The first block included gender, Extraversion, Agreeableness, Conscientiousness, Emotional Stability, and Openness to Experience. In the second block, we entered concrete, analytical, symbolic, and emotional aesthetic judgment styles. The results of the regression analysis are presented in Table 3. The Big Five in model 1 explained a significant proportion of variance in art interest, *R^2^* = .16, *F*(6, 246) = 7.74, *p* < .001. In the first model, gender, Emotional Stability, and Openness to Experience emerged as significant positive predictors of art interest. The second model was also significant, *R^2^* = .31, *F*(10, 242) = 10.83, *p* < .001. In the second model, Emotional Stability, Openness to Experience, and symbolic judgment style emerged as significant positive predictors and concrete aesthetic judgment style emerged as significant negative predictor of art interest.

**Table 3:** Hierarchical Regression Analysis With Art Interest as Dependant Variable

Variable	β	*t*	*p*
Model 1			
Gender	.26	4.20	< .001
Extraversion	-.001	-0.02	.98
Agreeableness	-.003	-0.05	.96
Conscientiousness	-.06	-0.99	.33
Emotional Stability	.17	2.78	.01
Openness to Experience	.25	4.16	< .001
Model 2			
Gender	.18	3.18	.002
Extraversion	.01	0.17	.86
Agreeableness	.05	0.96	.34
Conscientiousness	-.08	-1.41	.16
Emotional Stability	.14	2.47	.01
Openness to Experience	.18	3.14	.002
Emotional AJS	.03	0.60	.55
Symbolic AJS	.31	4.63	< .001
Analytical AJS	.04	0.65	.52
Concrete AJS	-.19	-3.25	.001

*Note*. AJS = Aesthetic Judgment Style; Gender was coded as 0 (male) and 1 (female).

## Discussion

The current study aimed to examine the associations between basic personality traits, aesthetic judgment styles, and art interest. Based on previous findings, we made five *a priori* hypotheses. Overall, we predicted that Openness to Experience would be positively associated with general interest in art activities and advanced aesthetic judgment styles. On the contrary, Openness to Experience was hypothesized to be negatively correlated with less advanced styles in artistic judgment. Finally, art interest was hypothesized to be positively associated with emotional judgment style (as an advanced style), while negatively related to concrete judgment style (as the least advanced artistic style; Pourhosein et al., 2017). Our analytic strategy included three consecutive analyses: descriptive analyses and sex differences, bivariate correlational analyses, and hierarchical regression analyses. All hypotheses were supported by data analysis.

In our descriptive and preliminary analyses, we found that women are more interested in art than men in the current sample. Considering the items assessing art interest, it can be concluded that women allocate a greater time to art and are more likely to visit art galleries. This corroborates previous work (see DiMaggio & Mukhtar, 2004). For example, McManus and Furnham (2006) found that men scored lower on overall aesthetic activities, literature, performance arts. Men scored significantly higher on concrete aesthetic judgment style, meaning that men are more likely to react to artworks using superficial qualities of those works. Another significant sex difference emerged in this study: Women scored higher on Agreeableness. This is in line with reported sex differences in the Big Five dimensions of personality in Iran (Afhami, Mohammadi-Zarghan, & Atari, 2017) and across cultures (Schmitt, Realo, Voracek, & Allik, 2008). The positive correlation between age and symbolic and analytical aesthetic judgment styles are in line with Pourhosein et al. (2017). These findings suggest that older individuals are more likely leave logical and conventional comments on works of art. Furthermore, older individuals in the current sample were more likely to be interested in abstract ideas and symbolic elements of the arts. Finally, age was positively associated with Conscientiousness. This is also consistent with previous works. For example, McCrae et al. (1999) used convenience samples from Germany, Italy, Portugal, Croatia, and Korea and found that self-reports of Conscientiousness was higher in older participants. More recently, Donnellan and Lucas (2008) found that among individuals between 20 and 30 (the majority of the participants in the current study), age was positively associated with Conscientiousness.

In our correlational analyses, we found Openness to Experience to be positively related to interest in art activities and emotional aesthetic judgment style. This is generally in line with the notion that aesthetics is an important facet of Openness to Experience, even when Openness to Experience is measured using ultra short measures. Those individuals who score higher on Openness to Experience are generally more imaginative, creative, and art-appreciating. These findings also suggest that open individuals are more likely to produce affective statements and comments when exposed to a piece of art. Furthermore, open individuals feel emotionally connected to the artwork. In addition, those who have more advanced styles of responding to art are more interested in art activities (versus those who use concrete style of artistic judgment).

The regression analyses suggested that sex, Emotional Stability, and Openness to Experience predict general interest in art, when all personality dimensions are simultaneously entered into the regression analysis. When aesthetic judgment styles were entered into the equations, sex, Emotional Stability, and Openness to Experience remained significant predictors of the dependant variable, and symbolic style (positively) and concrete style (negatively) emerged as significant predictors of art interest. The positive role of Emotional Stability in prediction of art interest is not consistent with positive correlations between Neuroticism (the low pole of Emotional Stability) and overall aesthetic activity (McManus & Furnham, 2006). However, the present findings are in line with the inverse relationship between Neuroticism and aesthetic fluency (Silvia, 2007). These differences may be attributable to the conceptual differences between “overall aesthetic activity”, “aesthetic fluency”, and “art interest”. Other studies (Furnham & Chamorro-Premuzic, 2004) have reported null relationships between Neuroticism and art interests. Alternatively, cultural differences may account for this inconsistency. Future research could replicate the associations between Emotional Stability and different aspects of aesthetic experience to get a clear image. These findings highlight the role of aesthetic judgment *style* to predict individuals’ general interest in art-related activities and artistic experiences. Regression analyses showed more than fourth of the variance could be accounted for by personality and aesthetic judgment styles.

One of the important implications of the present work is the utility of aesthetic judgment styles in predicting art interest. This can potentially be of interest to educational psychologists for art education. Taking aesthetic judgment styles as fixed, trait-like variables would suggest that certain people would be more interested in art. Of course, there is no reason to assume that these styles cannot be altered. Further, it might be possible to manipulate these “aesthetic judgment styles” using experimental paradigms (see Oyserman, Sorensen, Reber, & Chen, 2009). It is also useful to examine how such styles of aesthetic judgement can influence ratings of artworks in museum settings. For example, aesthetic judgment styles might moderate the fluency-pleasure link (see Graf & Landwehr, 2015). Therefore, it is important for future research to integrate different models of aesthetic preferences to better understand the cognitive, emotional, and personality correlates of general interest in art as well as contextual factors that contribute to aesthetic liking and pleasure when one is exposed to art.

This study has important findings. First, this is among the first studies to explore the additional value of the four-factor model of aesthetic judgment (Bahrami-Ehsan et al., 2015) in art settings. While Swami and Furnham (2014) suggested that aesthetic judgment as ability is no longer the focus of experimental aesthetics researchers, the present study highlighted the strong predictive role of aesthetic judgment “style” as an individual difference variable. Second, we used a non-Western sample. Most of the psychological studies on art and aesthetic experience have been conducted in Western societies, so the present findings may help expand the cross-cultural perspective in this line of research. Third, in addition to the main results, this study has important implications. For example, few studies have examined sex differences in the Big Five in Iran. Additionally, this is the first study to examine sex differences in aesthetic judgment styles.

The present study’s limitations are worth noting. First, we used cross-sectional data and correlational analyses. Therefore, the direction of the influences cannot be assured. For example, it is possible that art interest gradually influences and forms one’s aesthetic judgment style. Alternatively, one’s repetitive exposure to art can influence self-reports of Openness to Experiences. Future research could beneficially use longitudinal designs to overcome the methodological limitations of cross-sectional sampling and correlational analyses. Second, we used a college sample. It is important for future research to replicate and extend these findings in community samples. Third, we used a short measure to assess the Big Five (Gosling et al., 2003). Future research can use more up-to-date measures for assessment of the Big Five (Soto & John, in press). Fourth, we examined individuals’ interest in art in general. Yet, future research can examine interest in specific arts or in specific art domains. Finally, personality dimensions and aesthetic judgment styles may be used in future research to predict artistic preferences.

In conclusion, the current study showed that art interest was significantly associated with a number of individual difference factors, particularly Openness to Experience, Emotional Stability, and aesthetic judgment styles. As Chamorro-Premuzic, Reimers, Hsu, and Ahmetoglu (2009) noted, such results have a number of practical implications for wider society. For example, knowing the patterns of associations between individual difference variables and art-related activities can prove useful in order to promote art in the community and for its use in therapy.

## References

[r1] Afhami R. Mohammadi-Zarghan S. Atari M. (2017). Self-Rating of Religiosity (SRR) in Iran: Validity, reliability, and associations with the Big Five. Mental Health, Religion & Culture, 20, 879-887. doi:. 10.1080/13674676.2017.1313825

[r2] Atari M. Barbaro N. Sela Y. Shackelford T. K. Chegeni R. (2017). The Big Five personality dimensions and mate retention behaviors in Iran. Personality and Individual Differences, 104, 286–290. doi:. 10.1016/j.paid.2016.08.029

[r3] Atari M. Yaghoubirad M. (2016). The Big Five personality dimensions and mental health: The mediating role of alexithymia. Asian Journal of Psychiatry, 24, 59–64. doi:. 10.1016/j.ajp.2016.08.008 27931909

[r4] Axelsson Ö. (2007). Individual differences in preferences for photographs. Psychology of aesthetics, creativity, and the arts, 1, 61–72. doi:. 10.1037/1931-3896.1.2.61

[r5] Bahrami-Ehsan H. Mohammadi-Zarghan S. Atari M. (2015). Aesthetic judgment style: Conceptualization and scale development. International Journal of Arts, 5, 33–39. doi:. 10.5923/j.arts.20150502.01

[r6] Chamorro-Premuzic T. Furnham A. (2004). Art judgement: A measure related to both personality and intelligence? Imagination, Cognition and Personality, 24, 3–24. doi:. 10.2190/U4LW-TH9X-80M3-NJ54

[r7] Chamorro-Premuzic T. Reimers S. Hsu A. Ahmetoglu G. (2009). Who art thou? Personality predictors of artistic preferences in a large UK sample: The importance of openness. British Journal of Psychology, 100, 501–516. doi:. 10.1348/000712608X366867 19026107

[r8] Chatterjee A. Widick P. Sternschein R. Smith W. B. Bromberger B. (2010). The assessment of art attributes. Empirical Studies of the Arts, 28, 207–222. doi:. 10.2190/EM.28.2.f

[r9] De SmedtJ.De CruzH. (2010). Toward an integrative approach of cognitive neuroscientific and evolutionary psychological studies of art. Evolutionary Psychology, 8, 695–719. doi:. 10.1177/14747049100080041122947828

[r10] DeYoung C. G. Quilty L. C. Peterson J. B. Gray J. R. (2014). Openness to experience, intellect, and cognitive ability. Journal of Personality Assessment, 96, 46–52. doi:. 10.1080/00223891.2013.806327 23795918

[r11] DiMaggio P. Mukhtar T. (2004). Arts participation as cultural capital in the United States, 1982–2002: Signs of decline? Poetics, 32, 169–194. doi:. 10.1016/j.poetic.2004.02.005

[r12] Donnellan M. B. Lucas R. E. (2008). Age differences in the Big Five across the life span: Evidence from two national samples. Psychology and Aging, 23, 558–566. doi:. 10.1037/a0012897 18808245 PMC2562318

[r13] Eysenck H. J. Castle M. (1971). Comparative study of artists and non artists on the Maitland Graves Design Judgement Tests. The Journal of Applied Psychology, 55, 389–392. doi:. 10.1037/h0031469

[r14] Feist G. J. (1998). A meta-analysis of personality in scientific and artistic creativity. Personality and Social Psychology Review, 2, 290–309. doi:. 10.1207/s15327957pspr0204_5 15647135

[r15] Frois J. P. Eysenck H. (1995). The visual aesthetic sensitivity test applied to Portuguese children and fine arts students. Creativity Research Journal, 8, 277–284. doi:. 10.1207/s15326934crj0803_6

[r16] Furnham A. Chamorro-Premuzic T. (2004). Personality, intelligence, and art. Personality and Individual Differences, 36, 705–715. doi:. 10.1016/S0191-8869(03)00128-4

[r17] Gosling S. D. Rentfrow P. J. Swann W. B. (2003). A very brief measure of the Big-Five personality domains. Journal of Research in Personality, 37, 504–528. doi:. 10.1016/S0092-6566(03)00046-1

[r18] Graf L. K. Landwehr J. R. (2015). A dual-process perspective on fluency-based aesthetics: The pleasure-interest model of aesthetic liking. Personality and Social Psychology Review, 19, 395–410. doi:. 10.1177/1088868315574978 25742990

[r19] Graves, M. (1948). *Design Judgement Test.* San Antonio, TX, USA: Psychological Corporation.

[r20] Habibi A. Damasio A. (2014). Music, feelings, and the human brain. Psychomusicology: Music, Mind, and Brain, 24, 92–102. doi:. 10.1037/pmu0000033

[r21] John, O. P., Naumann, L. P., & Soto, C. J. (2008). Paradigm shift to the integrative Big Five trait taxonomy: History, measurement, and conceptual issue. In O. P. John, R. W. Robins, & L. A. Pervin (Eds.), *Handbook of personality: Theory and research* (pp. 114–158). New York, NY, USA: Guilford.

[r22] Leder H. Belke B. Oeberst A. Augustin D. (2004). A model of aesthetic appreciation and aesthetic judgments. British Journal of Psychology, 95, 489–508. doi:. 10.1348/0007126042369811 15527534

[r23] Loevinger, J. (1976). *Ego development: Conceptions and theories*. San Francisco, CA, USA: Jossey-Bass.

[r24] Maddi S. R. Berne N. (1964). Novelty of productions and desire for novelty as active and passive forms of the need for variety. Journal of Personality, 32, 270–277. doi:. 10.1111/j.1467-6494.1964.tb01340.x 14155502

[r25] McCrae R. R. (2007). Aesthetic chills as a universal marker of openness to experience. Motivation and Emotion, 31, 5–11. doi:. 10.1007/s11031-007-9053-1

[r26] McCrae R. R. Costa P. T. de Lima M. P. Simões A. Ostendorf F. Angleitner A. Chae J. H. (1999). Age differences in personality across the adult life span: Parallels in five cultures. Developmental Psychology, 35, 466–477. doi:. 10.1037/0012-1649.35.2.466 10082017

[r27] McManus I. C. Furnham A. (2006). Aesthetic activities and aesthetic attitudes: Influences of education, background and personality on interest and involvement in the arts. British Journal of Psychology, 97, 555–587. doi:. 10.1348/000712606X101088 17018189

[r28] Myszkowski N. Storme M. Zenasni F. (2016). Order in complexity: How Hans Eysenck brought differential psychology and aesthetics together. Personality and Individual Differences, 103, 156–162. doi:. 10.1016/j.paid.2016.04.034

[r29] Myszkowski N. Storme M. Zenasni F. Lubart T. (2014). Is visual aesthetic sensitivity independent from intelligence, personality and creativity? Personality and Individual Differences, 59, 16–20. doi:. 10.1016/j.paid.2013.10.021

[r30] Nusbaum E. C. Silvia P. J. (2011). Are openness and intellect distinct aspects of openness to experience? A test of the O/I model. Personality and Individual Differences, 51, 571–574. doi:. 10.1016/j.paid.2011.05.013

[r31] Osberg T. M. (1987). The convergent and discriminant validity of the Need for Cognition Scale. Journal of Personality Assessment, 51, 441–450. doi:. 10.1207/s15327752jpa5103_11 16372844

[r32] Oshio A. Abe S. Cutrone P. Gosling S. D. (2013). Big Five content representation of the Japanese version of the Ten-Item Personality Inventory. Psychology, 4, 924–929. doi:. 10.4236/psych.2013.412133

[r33] Oyserman D. Sorensen N. Reber R. Chen S. X. (2009). Connecting and separating mind-sets: Culture as situated cognition. Journal of Personality and Social Psychology, 97, 217–235. doi:. 10.1037/a0015850 19634972

[r34] Pourhosein R. Mohammadi-Zarghan S. Soufiabadi M. Atari M. (2017). Ego development and aesthetic judgment styles in Iranian adults. Psychological Thought, 10, 80–89. doi:. 10.5964/psyct.v10i1.213

[r35] Rawlings D. Barrantes-Vidal N. Furnham A. (2000). Personality and aesthetic preference in Spain and England: Two studies relating sensation seeking and openness to experience to liking for paintings and music. European Journal of Personality, 14, 553–576. doi:. 10.1002/1099-0984(200011/12)14:6<553::AID-PER384>3.0.CO;2-H

[r36] Reber R. Schwarz N. Winkielman P. (2004). Processing fluency and aesthetic pleasure: Is beauty in the perceiver’s processing experience? Personality and Social Psychology Review, 8, 364–382. doi:. 10.1207/s15327957pspr0804_3 15582859

[r37] Saucier G. (1992). Openness versus intellect: Much ado about nothing? European Journal of Personality, 6, 381–386. doi:. 10.1002/per.2410060506

[r38] Saucier G. (1994). Trapnell versus the lexical factor: More ado about nothing? European Journal of Personality, 8, 291–298. doi:. 10.1002/per.2410080406

[r39] Schmitt D. P. Realo A. Voracek M. Allik J. (2008). Why can’t a man be more like a woman? Sex differences in Big Five personality traits across 55 cultures. Journal of Personality and Social Psychology, 94, 168–182. doi:. 10.1037/0022-3514.94.1.168 18179326

[r40] Silvia P. J. (2006). Artistic training and interest in visual art: Applying the appraisal model of aesthetic emotions. Empirical Studies of the Arts, 24, 139–161. doi:. 10.2190/DX8K-6WEA-6WPA-FM84

[r41] Silvia P. J. (2007). Knowledge-based assessment of expertise in the arts: Exploring aesthetic fluency. Psychology of Aesthetics, Creativity, and the Arts, 1, 247–249. doi:. 10.1037/1931-3896.1.4.247

[r42] Silvia P. J. Nusbaum E. C. (2011). On personality and piloerection: Individual differences in aesthetic chills and other unusual aesthetic experiences. Psychology of Aesthetics, Creativity, and the Arts, 5, 208–214. doi:. 10.1037/a0021914

[r43] Soto C. J. John O. P. (in press). The next Big Five Inventory (BFI-2): Developing and assessing a hierarchical model with 15 facets to enhance bandwidth, fidelity, and predictive power. Journal of Personality and Social Psychology. 10.1037/pspp0000096 27055049

[r44] Stallings B. Anderson F. (1969). Some characteristics and correlates of the Meier Art Test of Aesthetic Perception under two systems of scoring. Journal of Educational Measurement, 6, 179–185. doi:. 10.1111/j.1745-3984.1969.tb00676.x

[r45] Storme M. Tavani J. L. Myszkowski N. (2016). Psychometric properties of the French Ten-Item Personality Inventory (TIPI). Journal of Individual Differences, 37, 81–87. doi:. 10.1027/1614-0001/a000204

[r46] Swami, V., & Furnham, A. (2014). Personality and aesthetic experiences. In P. P. L. Tinio & J. K. Smith (Eds.), *The Cambridge handbook of the psychology of aesthetics and the arts* (pp. 540-561). Cambridge, United Kingdom: Cambridge University Press.

[r47] Swami V. Stieger S. Pietschnig J. Voracek M. (2010). The disinterested play of thought: Individual differences and preference for surrealist motion pictures. Personality and Individual Differences, 48, 855–859. doi:. 10.1016/j.paid.2010.02.013

[r48] Tschacher W. Bergomi C. Tröndle M. (2015). The Art Affinity Index (AAI): An instrument to assess art relation and art knowledge. Empirical Studies of the Arts, 33, 161–174. doi:. 10.1177/0276237415594709

